# Brugada Syndrome and Sudden Cardiac Death: An Electrocardiographic History

**DOI:** 10.5811/cpcem.19477

**Published:** 2024-08-02

**Authors:** Mark L. Moubarek, Gordon X. Wong, James S. Ford

**Affiliations:** * University of California, Davis Health, Department of Emergency Medicine, Sacramento, California; † University of California, Davis Health, Department of Internal Medicine, Division of Cardiovascular Medicine, Sacramento, California; ‡ University of California, San Francisco, Department of Emergency Medicine, San Francisco, California

**Keywords:** *Brugada syndrome*, *sudden cardiac death*, *cardiac arrest*, *coved ST-segment elevation*, *saddleback ST-segment elevation*

## Abstract

**Case Presentation:**

A 22-year-old male with a history of anti-neutrophil cytoplasmic antibody vasculitis, renal transplant, hypertension, and no known family history of sudden cardiac death suffered a witnessed cardiac arrest. An initial rhythm strip recorded by emergency medical services revealed ventricular fibrillation. Return of spontaneous circulation was achieved after three rounds of cardiopulmonary resuscitation, defibrillation, and intravenous epinephrine. The patient was brought to the emergency department and admitted to the intensive care unit. He was diagnosed with Brugada syndrome, and an automatic implantable cardioverter-defibrillator (AICD) was placed after discharge.

**Discussion:**

Brugada syndrome is characterized electrocardiographically by ≥2 millimeters (mm) ST-segment elevation in leads V_1_–V_2_ with either “coved type” (type 1) or “saddleback” (type 2) ST-segment morphology, or ≤2 mm ST-segment elevation in V_1_–V_2_ with either “coved” or “saddleback” morphology (type 3). The absence of these patterns on isolated electrocardiograms (ECG) does not exclude the diagnosis, as dynamic fluctuations in ECG patterns are well-documented and can be induced by various physiologic stressors. This case provides an uncommon, complete electrocardiographic history of Brugada syndrome, from out-of-hospital cardiac arrest to AICD placement and depicts dynamic fluctuations between Brugada patterns and normal ECGs. This highlights the importance of serial ECGs in diagnosis, as sudden cardiac death is often the first or only presentation of Brugada syndrome.

CPC-EM Capsule What do we already know about this clinical entity?

*The absence of classic Brugada patterns (“coved” or “saddleback” ST-segment elevations) on isolated electrocardiograms (ECG) does not exclude the diagnosis.*

What is the major impact of the images?

*Images depict dynamic changes between Brugada patterns and normal ECGs, from cardiac arrest to automatic implantable cardioverter-defibrillator placement.*

How might this improve emergency medicine practice?

*Serial ECGs are important in diagnosis, as sudden cardiac death is often the first or only presentation of Brugada syndrome.*

## CASE PRESENTATION

**Image 1.**
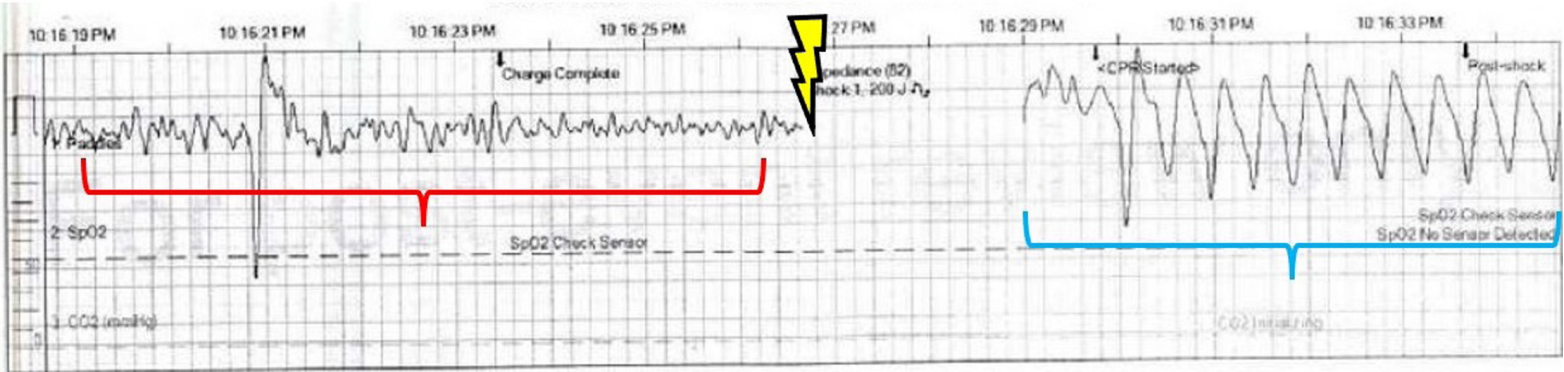
Rhythm strip performed by emergency medical services showing ventricular fibrillation (red bracket) followed by defibrillation (lightning symbol), with conversion to a wide-complex tachycardia consistent with ventricular tachycardia (blue bracket).

**Image 2.**
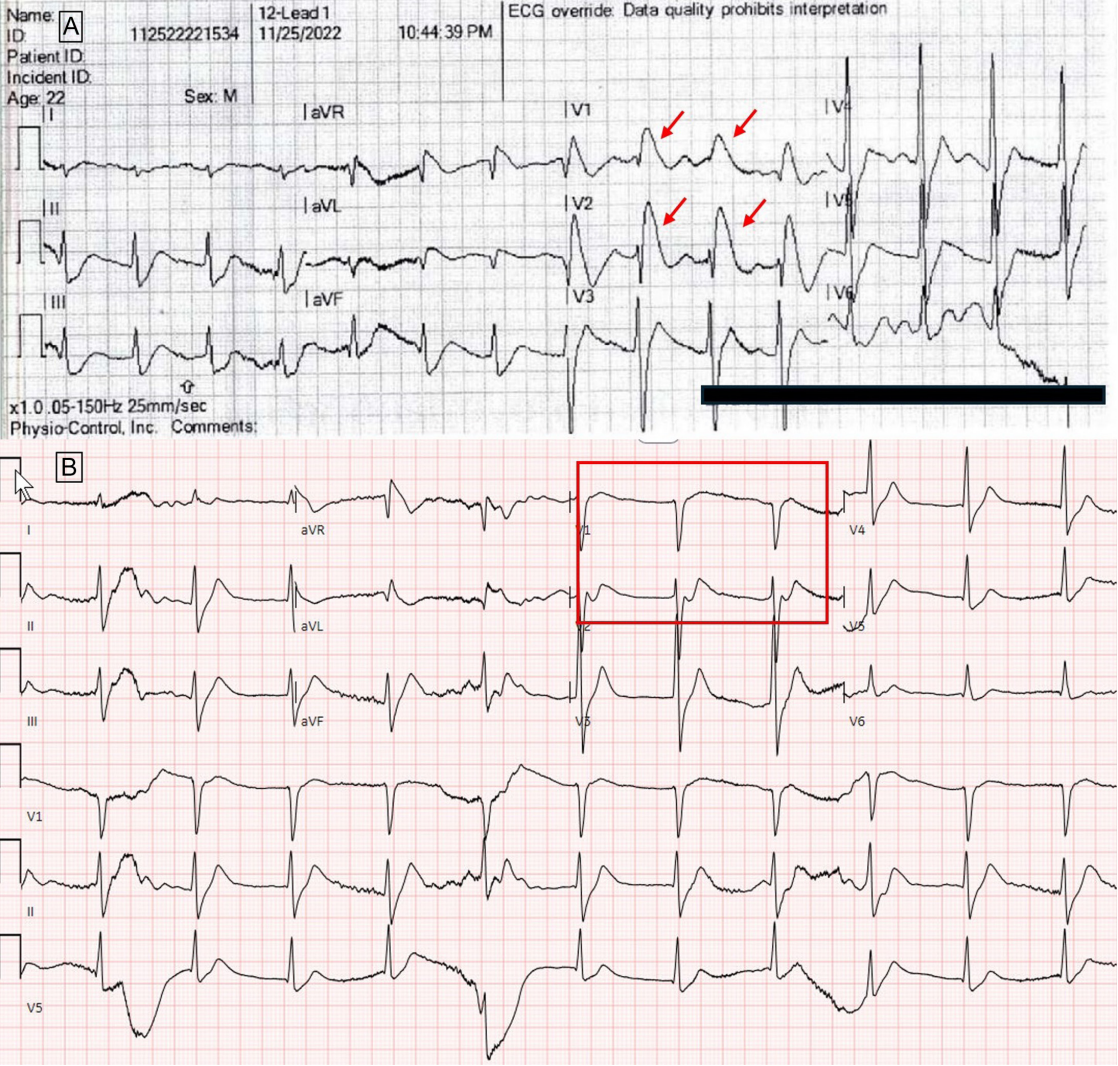
A. Electrocardiogram performed by emergency medical services prior to arrival to the emergency department, showing Brugada pattern (type 1) ST-segment elevation in leads V_1_–V_2_ (red arrows). B. Initial ECG performed in the ED, with resolution of Brugada pattern ST-segment elevations (red box).

A 22-year-old male, with a history of anti-neutrophil cytoplasmic antibody vasculitis, renal transplant, hypertension, and no known family history of sudden cardiac death, suffered a witnessed out-of-hospital cardiac arrest, receiving bystander cardiopulmonary resuscitation (CPR). An initial electrocardiogram (ECG) rhythm strip in the field revealed ventricular fibrillation (VF) (Image 1). The patient achieved return of spontaneous circulation (ROSC) after three milligrams (mg) of intravenous (IV) epinephrine, 450 mg IV amiodarone, and three rounds of CPR and defibrillation. He was intubated in the field. A post-ROSC ECG demonstrated Brugada type 1 ST-segment elevation in V_1_–V_2_ (Image 2A).^1^ In the emergency department (ED) an ECG showed dynamic resolution of the Brugada pattern (Image 2B).

The patient was given calcium gluconate empirically for the treatment of presumptive hyperkalemia, given his history of renal transplant. In the ED, labs were notable for a pH of 6.90 (reference range 7.35–7.40); partial pressure of carbon dioxide 67 millimeters of mercury (mm Hg) (35–45 mm Hg), bicarbonate 13 milliequivalents per liter (mEq/L) (22–28 mEq/L), and potassium 2.4 mEq/L (3.5–5.2 mEq/L). Four hours later, pH and potassium normalized without further intervention. The patient briefly required a norepinephrine infusion for low blood pressure and was given empiric broad spectrum antibiotics to cover for possible sepsis. Antibiotics were discontinued after a negative infectious workup.

**Image 3.**
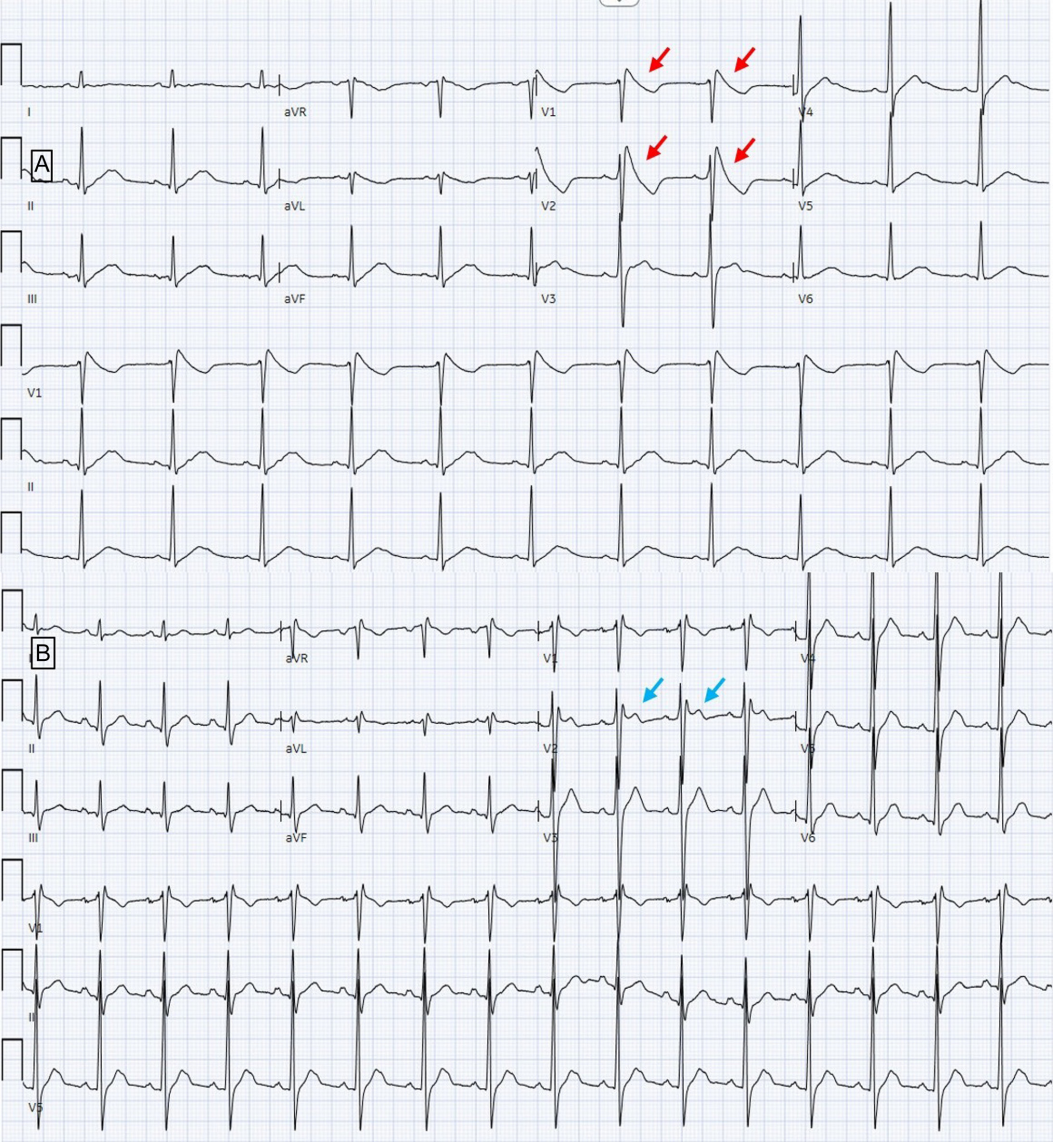
A. Electrocardiogram (ECG) performed approximately 60 hours post initial cardiac arrest, again with down-sloping ST-segment (Type 1) elevations in leads V_1_–V_2_ (red arrows). B. ECG performed 10 days after initial cardiac arrest showing saddleback ST-segment elevation <2 mm (type 3) in lead V_2_ (blue arrows).

An echocardiogram and computed tomography of the head, chest, abdomen, and pelvis were unremarkable. The patient was admitted to the intensive care unit and underwent targeted temperature management. An ECG from hospital day five re-demonstrated a type 1 Brugada pattern (Image 3A), and an ECG from hospital day 10 showed a type 3 Brugada pattern (Image 3B).^2^ Given the re-demonstration of Brugada patterns despite normalization of laboratory derangements and no other identified cause of cardiac arrest, he was diagnosed with Brugada syndrome. No formal electrophysiologic testing was performed. He was discharged on hospital day 28 with a LifeVest (Zoll Medical Corporation, Pittsburgh, PA) after a near-complete physical and neurologic recovery, and he underwent outpatient automatic-implantable-cardioverter-defibrillator (AICD) placement. Genetic testing performed later as an outpatient was inconclusive.

## DISCUSSION

Brugada syndrome is characterized electrocardiographically by ≥2 mm ST-segment elevation in leads V_1_–V_2_ with either “coved type” (type 1) or “saddleback” (type 2) ST-segment morphology, or ≤2 mm ST-segment elevation in V_1_–V_2_ with either “coved” or “saddleback” morphology (type 3).^1^ ^,^ ^2^ The absence of these patterns on isolated ECGs does not exclude the diagnosis, as dynamic fluctuations in ECG patterns are well-documented and can occur in response to medications, fever, exercise or other stressors ^1^ ^,^ ^3^ While this patient did not undergo formal electrophysiologic testing, established diagnostic criteria do not necessitate this, and its utility is questionable in VF-survivors.^2^ Similarly, this patient’s genetic testing was inconclusive; only 10–30% of patients have been successfully genotyped, owing to the broad heterogeneity and complexity of underlying genetic risk factors that can predispose an individual to Brugada syndrome.^2^

This case provides an uncommon, complete electrocardiographic history of Brugada syndrome, from out-of-hospital cardiac arrest to AICD placement and depicts classic dynamic fluctuations between Brugada patterns and normal rhythms on ECG. This highlights the importance of serial ECGs in diagnosis, as sudden cardiac death is often the first or only presentation of Brugada syndrome.^4^
